# Determination of Personalized Asthma Triggers From Multimodal Sensing and a Mobile App: Observational Study

**DOI:** 10.2196/14300

**Published:** 2019-06-27

**Authors:** Revathy Venkataramanan, Krishnaprasad Thirunarayan, Utkarshani Jaimini, Dipesh Kadariya, Hong Yung Yip, Maninder Kalra, Amit Sheth

**Affiliations:** 1 Ohio Center of Excellence in Knowledge-enabled Computing Wright State University Dayton, OH United States; 2 Dayton Children's Hospital Dayton, OH United States

**Keywords:** personalized digital health, medical internet of things, asthma management, patient-generated health data, pediatric asthma, asthma control, medication adherence, childhood asthma, understanding and treatment of asthma

## Abstract

**Background:**

Asthma is a chronic pulmonary disease with multiple triggers. It can be managed by strict adherence to an asthma care plan and by avoiding these triggers. Clinicians cannot continuously monitor their patients’ environment and their adherence to an asthma care plan, which poses a significant challenge for asthma management.

**Objective:**

In this study, pediatric patients were continuously monitored using low-cost sensors to collect asthma-relevant information. The objective of this study was to assess whether kHealth kit, which contains low-cost sensors, can identify personalized triggers and provide actionable insights to clinicians for the development of a tailored asthma care plan.

**Methods:**

The kHealth asthma kit was developed to continuously track the symptoms of asthma in pediatric patients and monitor the patients’ environment and adherence to their care plan for either 1 or 3 months. The kit consists of an Android app–based questionnaire to collect information on asthma symptoms and medication intake, Fitbit to track sleep and activity, the Peak Flow meter to monitor lung functions, and Foobot to monitor indoor air quality. The data on the patient’s outdoor environment were collected using third-party Web services based on the patient’s zip code. To date, 107 patients consented to participate in the study and were recruited from the Dayton Children’s Hospital, of which 83 patients completed the study as instructed.

**Results:**

Patient-generated health data from the 83 patients who completed the study were included in the cohort-level analysis. Of the 19% (16/83) of patients deployed in spring, the symptoms of 63% (10/16) and 19% (3/16) of patients suggested pollen and particulate matter (PM2.5), respectively, to be their major asthma triggers. Of the 17% (14/83) of patients deployed in fall, symptoms of 29% (4/17) and 21% (3/17) of patients suggested pollen and PM2.5, respectively, to be their major triggers. Among the 28% (23/83) of patients deployed in winter, PM2.5 was identified as the major trigger for 83% (19/23) of patients. Similar correlations were not observed between asthma symptoms and factors such as ozone level, temperature, and humidity. Furthermore, 1 patient from each season was chosen to explain, in detail, his or her personalized triggers by observing temporal associations between triggers and asthma symptoms gathered using the kHealth asthma kit.

**Conclusions:**

The continuous monitoring of pediatric asthma patients using the kHealth asthma kit generates insights on the relationship between their asthma symptoms and triggers across different seasons. This can ultimately inform personalized asthma management and intervention plans.

## Introduction

### Background

Asthma is a chronic inflammatory lung disease affecting 26 million people in the United States, of which 6 million are children [1]. It is a multifactorial disease, with exposure to different triggers manifesting as symptoms of varying intensities, which demands a personalized diagnosis and management plan [2]. Timely feedback and intervention are not possible with infrequent clinical visits because most of the asthma-exacerbating factors are in the patient’s environment and are not tracked meticulously [3,4] or because of the lack of medication adherence [5]. Continuous tracking and assessment of a patient’s condition, environment, and adherence to a prescribed care plan can improve asthma control and quality of life [6].

Although many studies have shown the effectiveness of continuous monitoring, only a few are being evaluated to benefit traditional health care practices [7,8]. Propeller Health [9] provides personalized alerts based on inhaler usage and location to primarily improve medication adherence. ENVIROFI [10] and azma.com [11] send a notification to subscribed users when the outdoor environment forecast is poor. Chu et al [12] developed a ubiquitous warning system that sends alerts to health care providers based on a patient’s location if the outdoor environment is poor. Finkelstein et al [13] developed a Web-based approach that captures the patient’s forced vital capacity test and asthma symptoms and sends alerts to hospitals when these parameters are abnormal. AsthmaGuide [14], a home management ecosystem, enables doctors to observe the correlation between symptoms and environmental data. They have classified wheezing sounds as asthmatic wheezing and nonasthmatic wheezing. They also send personalized alerts to patients based on pollen and air quality forecast, but no causal relationships are identified. The existing studies analyze the data pertaining to the outdoor environment to identify the causes of asthma symptoms or improve medication adherence, but these studies have not used a large cohort of pediatric patients in a clinical setting and have not monitored and analyzed a comprehensive set of factors such as the lung function measurements, activity limitation, and data pertaining to the indoor environment to personalize asthma management plan.

### kHealth Asthma Framework

The researchers at Knoesis (Ohio Center of Excellence in BioHealth and Innovations) developed kHealth [15] (Multimedia Appendix 1), a multisensory framework for continuous monitoring of patients’ health signals and environmental data. The kHealth kit collects multimodal data using low-cost sensors and mobile apps, and kHealth methodology analyzes them for personalized health management plan. kHealth asthma [16] is an adaptation of kHealth framework and methodology for asthma, which monitors the pediatric patients receiving asthma care at the Dayton Children’s Hospital (DCH). The motivations behind kHealth asthma kit are to (1) identify the personalized triggers from the comprehensive data collected by kHealth kit and rank their influence on asthma, which the paper focuses on, and (2) provide actionable insights to clinicians for better decision making related to asthma management based on specific patient data. It is designed to assist patients in self-monitoring and self-appraisal of asthma care, with an intent to incorporate self-management, prediction, and intervention [17]. This paper presents a cohort-level analysis of patients deployed over the entire year to evaluate the ability of low-cost sensors in identifying the major triggers for their asthma symptoms. Specifically, 1 patient from each season was chosen to illustrate the personalized determination of triggers by gathering anecdotal evidence.

## Methods

The kHealth asthma framework consists of the kHealth kit, kHealth cloud, and kHealth Dashboard. The study design, including these components, their use for data collection, and the data analysis are discussed below. Other applications for which kHealth has been adapted include postbariatric surgery monitoring, postsurgery monitoring of acute decompensated heart failure, and dementia.

### kHealth Components

#### kHealth Kit

**Figure 1:**
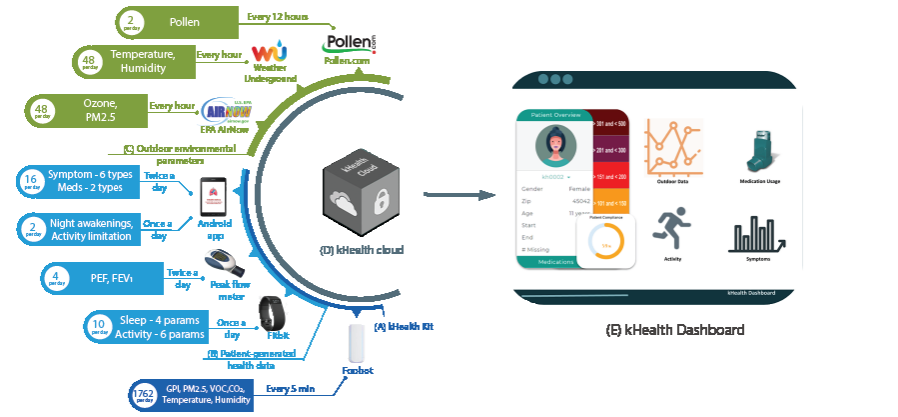
The kHealth framework with the kHealth kit, kHealth cloud, and kHealth Dashboard, showing the frequency of data collection, the number of parameters collected, and the total number of data points collected per day per patient. (A) Dark blue; the kHealth kit components that are given to patients. (B) Light blue; the kHealth kit components that collect patient-generated health data. (C) Green; the outdoor environmental parameters and their sources. (D) The kHealth cloud (gray). (E) The kHealth Dashboard. Throughout the kHealth ecosystem, all data are anonymized and associated with respective randomly assigned patient IDs. FEV1: forced expiratory volume in 1 second; PEF: peak expiratory flow; PM2.5: particulate matter.

The kHealth kit components are shown in Figure 1. The list of components are as follows: (1) tablet with an Android app; (2) Fitbit; (3) Peak Flow meter; (4) Foobot, the indoor air quality monitor; and (5) Web services to collect data on outdoor environment based on the patient’s zip code. The questionnaire presented by the kHealth Android app on the tablet collects the following data: (1) 6 types of symptoms: cough, wheeze, chest tightness, hard and fast breathing, cannot talk in full sentences, and nose opens wide [18]; (2) medication intake (rescue inhaler and controller medication) with yes or no option, (3) nighttime awakenings because of asthma symptoms, and (4) activity limitation because of asthma symptoms. The data on symptoms and medications are collected twice a day, and data on nighttime awakenings and activity limitation are collected once a day (Multimedia Appendix 2). Furthermore, Fitbit is used to collect more granular data for sleep and activity [19]. The lung function measurements (peak expiratory flow [PEF] and forced expiratory volume in 1 second [FEV_1_]) are recorded by the Microlife peak flow meter [20] twice every day. For a given patient’s zip code, outdoor environmental parameters are collected at different intervals—pollen is collected every 12 hours, whereas particulate matter (PM2.5), ozone, temperature, and humidity are collected every hour. Pollen is collected from pollen.com [21], PM2.5 and ozone from EPA AIRNow [22], and temperature and humidity from Weather Underground [23]. Foobot collects indoor temperature, humidity, PM2.5, volatile compounds, carbon dioxide, and global pollution index every 5 min.

#### Validation of Kit Components

Our tablet questionnaire is based on the Asthma Control Test (ACT) questionnaire [24] adapted for Android app and was developed under the supervision of the clinician (the question text was adjusted to make it user friendly). The questions were tested with the patients and iteratively refined using the preliminary work of the evaluation. While recruiting and consenting young patients, the nurse educates both the patient or guardian and the child on the correct way to use the kHealth kit. Given that the child is using kHealth kit under adult supervision and the sensors are reasonably robust, we expect the data to be reliable, and the parent and child team to be trustworthy. Our data and experience suggest that peak flow values measured by Peak Flow meter can vary dramatically in the 3 trials conducted before picking the maximum value. However, it was possible to distinguish normal state from asthmatic state using the max peak flow value so obtained. Previous studies have reported feasibility of peak flow measurement in 5-year-old children [25,26]. The outdoor environmental parameters are collected from EPA AIRNow, Weather Underground, and pollen.com, which are reputed sources, and already published works have used their data [27-29]. We have relied on existing feasibility studies for Fitbit [30,31], but we performed our own for Foobot [32]. In our recent data collection, all the components of the kHealth kit except Foobot (indoor air quality monitor) worked as advertised by the vendor. Although Foobot gave reliable results in our feasibility study, the device suffered electronic interference in the patient’s environment, leading to unreliable results.

#### kHealth Cloud

The multimodal data collected from various sources are brought together on the secure kHealth cloud store. The data on outdoor environment, indoor air quality, activity, and sleep are collected from their respective application programming interface (API) server and stored directly in the kHealth cloud. The data collected using the kHealth app, which includes the patient’s symptoms, medication intake, PEF, and FEV_1_ readings, are synced in real time with Firebase, a Google cloud database [33]. Firebase provides active data listener for client side, which offers data persistence over the network failure and resyncs to the cloud when the network is restored. Data security is maintained in Firebase using a set of data access rules and user authentication. Data synced to Firebase are then fetched and stored into the kHealth cloud. This process forms a pipeline for seamless and reliable data streaming from the kHealth app to the kHealth cloud. All data stored in the kHealth cloud are then made available to Knoesis researchers and clinicians for real-time analysis. Each patient’s identity is anonymized by the nurse coordinator who obtains the patient’s consent; no patient-identifiable data are stored anywhere in the kHealth framework.

#### kHealth Dashboard

**Figure 2:**
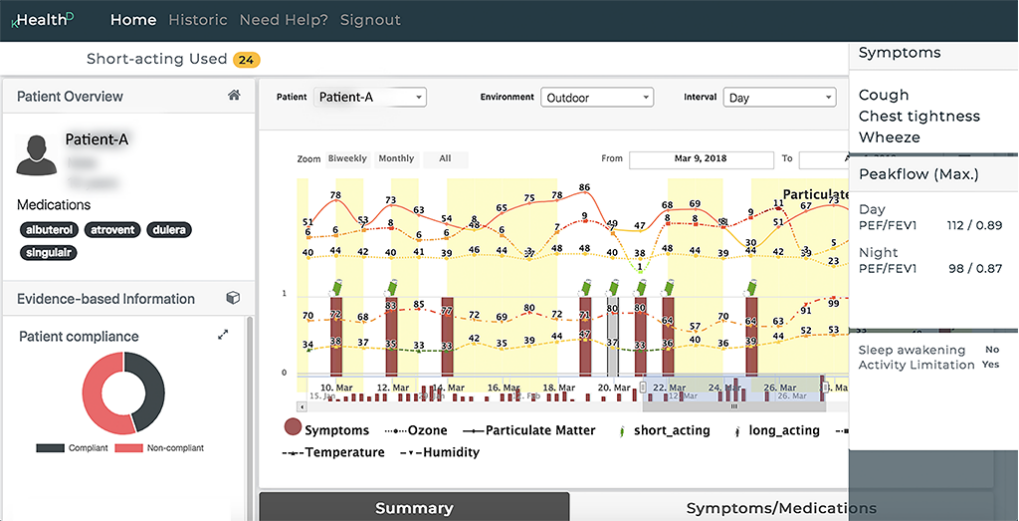
A screenshot of the kHealth Dashboard visualizing patient A’s data. FEV1: forced expiratory volume in 1 second; PEF: peak expiratory flow.

Because the kHealth kit collects multimodal data at different frequencies, integration and visualization of these data are essential to derive useful insights. kHealth Dashboard [34] (Figure 2) is a visualization and analysis tool designed for use by a clinician and a researcher to review individual and aggregated data and explore the potential association between patient’s asthma symptoms and their environments. With real-time data available in the kHealth cloud, kHealth Dashboard allows real-time monitoring of a patient’s asthma condition. This granularity of data presents the clinician with a better picture about patient’s asthma condition than in traditional episodic clinical visits (see Multimedia Appendix 1 for demo video).

### Study Design and Participants

The children within the age group of 5 to 17 years and diagnosed with asthma (through standard clinical procedures) by our clinician were recruited from the DCH. The research nurse practitioner, under the guidance of the clinician, approached the parent of the child to participate in our study. The parent, along with the child, was consented to participate in our study. The recruitment for the study was random, with the motive of maximizing participation and done on a first-come-first-serve basis. The tablet with Android app, Peak Flow meter, Fitbit, and Foobot were given to each patient, and based on the patient’s zip code, outdoor environmental data were collected. The part of data collection, which requires the patient to be actively involved (such as responding to Android app questionnaire and taking Peak Flow meter readings), was referred to as *active sensing*. The data collection from Fitbit, Foobot, and outdoor Web services, which do not require active patient involvement, was referred to as *passive sensing*.

Inclusion criteria [35] were children (1) diagnosed with asthma through standard clinical procedure by our clinician from DCH, (2) aged between 5 and 17 years, and (3) willing to participate in this study.

Of 107 patients, 24 patients were excluded from the analysis, as they did not complete the study, allowing us to analyze data from the remaining 83 patients. Both our National Institutes of Health (NIH) and National Institute of Child Health and Human Development (NICHD) proposal and our approved institutional review board (IRB) protocol explicitly indicate 2 populations: (1) consented patients and (2) a subgroup of patients who completed the evaluation (with adequate compliance in data collection). These 24 patients were ignored because they did not complete the study and did not provide adequate data for us to perform any meaningful analysis and offer sensible conclusions. We have explicitly mentioned in our approved IRB protocol that only the patients who consented and patients who completed the study will be included in our analysis. There has been no selection bias based on data provided by or collected from a patient in either the protocol or the study presented in this paper. No patients who completed the study were ignored from this analysis. Of the 83 patients who completed the study, 63 were recruited for a month, and 20 were recruited for a 3-month period. The 1-month study was designed to validate the efficacy of this method, and the 3-month study was included to obtain sufficient data and determine the association between various asthma-relevant factors and asthma symptoms. The enrollment of a patient for 1 or 3 months depended on their willingness.

This Health Insurance Portability and Accountability Act–compliant study has been approved by DCH’s IRB. Given the multifactorial nature of asthma, we sought to monitor as many variables as practically possible, subject to our constraints of practical implementation (eg, using technology that can be deployed at the patient’s home) and cost (our NIH and NICHD proposal and protocol called for purchasing 30 kits for proposed patient evaluation at approximately US $500 per kit).

Preliminary work for this effort was done in 2014 when mobile apps and low-cost sensors became viable [36]. When the NIH and NICHD proposal was written following the preliminary work, there were no reported efforts involving the use of multiple sensors (that can be used outside a clinical facility and at a patient’s home) and mobile apps (to record patient’s symptoms and collect sensor data). The wearables were just becoming popular, and the concept of patient-generated health data (PGHD) was relatively new. For the studies reporting on the collection and evaluation of personalized data for pediatric asthma patients, our study collects more extensive types of data at a higher frequency. Specifically, evaluation in the study by Merchant et al [37] involved 89 patients, with 2 types of data or parameters (medication and ACT score) observed for 12 months per patient, and Bender et al [38] involved 27 patients, with 1 parameter observed for 2 months. Our study involves 83 patients (63 patients for a 1-month duration and 20 patients for a 3-month duration), with 10 data types (not including Foobot and Fitbit), with each data type collected between 2 and 24 times a day.

### Study Procedures

Henceforth, the 6 asthma symptoms (cough, wheeze, chest tightness, hard and fast breathing, cannot talk in full sentences, and nose opens wide), nighttime awakenings, activity limitation, rescue medication intake, and abnormal PEF or FEV_1_ value will be collectively referred to as *asthma episodes*, a usage that is consistent with 2 previous studies [39,40]. Specifically, asthma can affect lung function and can manifest as lower values for PEF and FEV_1_ parameters [41]. Therefore, the reduction of PEF and FEV_1_ values beyond 1 standard deviation of the mean is treated as an episode of asthma. The duration of the seasons has been chosen to aid the analysis based on the historical pollen data. Although the deployments started in December 2016 and are still ongoing, the data only from December 2016 to February 2019 have been included in the analysis.

Maximum values of outdoor environmental data (ozone and PM2.5) over a day were considered to identify the correlation between triggers and asthma episodes. The healthy range for each outdoor parameter is as follows: 0 to 2.4 for pollen [42] and 0 to 50 for ozone and PM2.5 [43]. Any value above or below the healthy range on the day with asthma episodes, or the previous day, is counted as a contributor to the patient’s asthma episodes. We chose a 48-hour window because inflammation and late allergic reaction in asthma are characterized by prominent participation of eosinophils, and previous studies have demonstrated an exaggerated response in asthma patients up to 48 hours after exposure [44]. We analyzed concrete patient cases to obtain insights about asthma triggers, patient behavior, and their condition from evidence collected by kHealth Asthma technology in different seasons.

## Results

### Cohort-Level Analysis

Although we collected extensive Foobot data, the results on the influence of indoor environment were inconclusive because of deficiencies in our instructions such as (1) clarity on the placement of Foobot and (2) potential for electrical interference when some patients did not power the device as required by the manufacturer. In the study results recorded thus far, the outside environmental data provide the most reliable signals for asthma control. Furthermore, the sleep and activity data from Fitbit did not provide corroborative evidence for asthma signs because of several confounding factors (could be due to asthma symptoms or school routine or that Fitbit was not worn or powered). In contrast, self-reported data regarding activity limitation obtained through the Android app–based questionnaire proved to be more reliable. The results of cohort-level data analysis for all the completed patients (n=83) deployed in each season for the detection of a wide variety of triggers for asthma episodes using kHealth kit are shown in Table 1.

Among the 19% (16/83) of the patients deployed in spring, 63% (10/16) of the patients were affected by pollen, 16% (3/16) were affected by PM2.5, and 5% (1/16) were affected by pollen and PM2.5 (being present together). In addition, 17% (14/83) of the patients were deployed in fall, of which pollen turned out to be the major contributor for 29% (4/14), PM2.5 for 21% (3/14), and pollen and PM2.5 (being present together) for 14% (2/14). For 28% (23/83) of the patients deployed in winter, PM2.5 turned out to be the contributor for 83% (19/23). The 3-month deployments across 2 seasons enabled us to study the patient’s asthma in 2 different environments. For 29% (24/83) of the patients deployed across seasons, 50% (12/24) did not exhibit any symptoms. Moreover, 13% (3/24) indicated PM2.5 as their major trigger, 13% (3/24) indicated combination of pollen and PM2.5 to be the trigger, and 25% (6/24) of the patients had varying environment and too few symptoms to arrive at a conclusion. We observed meaningful (and useful according to our clinical partner) correlation between asthma symptoms and factors such as pollen and PM2.5. However, similar correlations were not observed for factors such as ozone level, temperature, and humidity. Personalized insights gathered using the kHealth kit are explained in detail in the Discussion section by choosing 1 patient from each season for illustration. The cohort-level and adherence statistics have already been presented in our previous study [45].

**Table 1:** Significant triggers captured by kHealth for each season at cohort level (N=83).

Season	n (%)	Pollen, %	PM2.5^a^, %	Ozone, %	Pollen and PM2.5, %	Temperature, %	No symptoms, %	Redeployment required, %
Spring	16 (19)	63	19	—^b^	6	—	12	—
Summer	6 (7)	17	33	—	33	—	17	—
Fall	14 (17)	29	21	—	14	—	36	—
Winter	23 (28)	—	83	4	—	9	4	—
Between seasons	24 (29)	—	12	—	33	—	50	25

^a^PM2.5: particulate matter.

^b^Not applicable.

### Personalized Analysis

**Figure 3:**
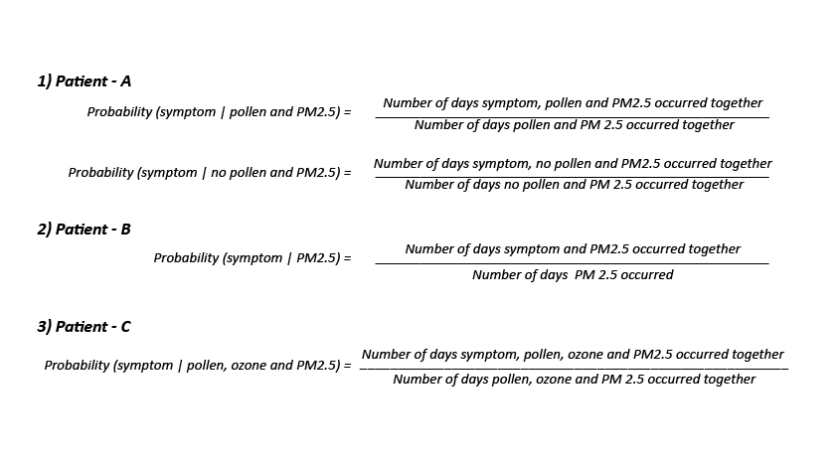
Formulae used to calculate the personalized triggers for patient-A, patient-B, and patient-C. PM2.5: particulate matter.

The probability of symptoms, given the triggers for patient A, is shown in Table 2. Patient A was monitored for 3 months from winter to spring; thus, the deployment duration is divided into 2, based on the presence and absence of pollen. The patient experienced symptoms in the presence of PM2.5, suggesting that PM2.5 was contributing to the patient’s asthma. In the presence of both pollen and PM2.5, the patient experienced higher number of severe symptoms such as chest tightness and nighttime awakenings, that is, the combination of pollen and PM2.5 aggravated patient’s asthma, as evidenced by cumulative increase in asthma symptoms. The rescue medication has also been included, as it could prevent or suppress the symptoms that could have occurred otherwise, such as wheezing. The Discussion section provides detailed patient information and calculations (see Figure 3 for formulas and Multimedia Appendix 3 for terms and definitions).

The probability of symptoms, given the triggers, for patient B, are shown in Table 3. Patient B was monitored in winter for 3 months when pollen was absent, and ozone was in a healthy range. The values suggest that PM2.5 was contributing to patient’s asthma, but the probabilities calculated are low as the patient took controller medication to reduce symptoms. The rescue medication is also included, as it can prevent or suppress the other symptoms. Discussion section provides detailed patient information and calculation (see Figure 3 for formulas and Multimedia Appendix 3 for terms and definitions).

The probability of symptoms, given the triggers for patient C, are shown in Table 4. Patient C was monitored for 36 days in fall when pollen, PM2.5, and ozone were in an unhealthy range. All the 3 triggers appear to be contributing to patient’s asthma. The patient was on an oral steroid that controlled the asthma episodes in the later part of the deployment, which explains the low probability values. The rescue medication is also included, as it can prevent or suppress the other symptoms that could have occurred. The Discussion section provides detailed patient information and calculation (see Figure 3 for formulas and Multimedia Appendix 3 for terms and definitions).

**Table 2:** Probability of symptoms, given the triggers, for patient A.

Symptoms	Probability (symptoms | pollen and PM2.5^a^)	Probability (symptoms | no pollen and PM2.5)
Cough	0.66	0.52
Wheeze	0.72	0.88
Chest tightness	0.28	0.12
Activity limitation	0.28	0.64
Nighttime awakenings	0.33	0.04
Rescue medication intake	0.55	0.48

^a^PM2.5: particulate matter.

**Table 3:** Probability of symptoms, given the triggers, for patient B.

Symptom	Probability (symptoms | PM2.5^a^)
Wheeze	0.51
Activity limitation	0.25
Nighttime awakenings	0.02
Rescue medication	0.54

^a^PM2.5: particulate matter.

**Table 4:** Probability of symptoms, given the triggers, for patient C.

Symptoms	Probability (symptoms | PM2.5^a^, pollen and ozone)
Cough	0
Wheeze	0.22
Activity limitation	0.11
Rescue medication intake	0.22

^a^PM2.5: particulate matter.

## Discussion

### Principal Findings

The kHealth kit is able to identify personalized triggers for each season for all the patients. As expected, pollen was the major contributor for the patients with asthma who were deployed in spring and fall. In winter, PM2.5 was the major contributor for most patients.

To illustrate the determination of personalized triggers and its dependence on the seasons, 1 patient with a relatively high number of asthma episodes was chosen for each season. Patient A’s deployment period straddled 2 seasons, winter and spring, which permitted the study of asthma in both the presence and absence of pollen. The other 3 patients were deployed exclusively within 1 season: patient B in winter, patient C in fall, and patient D in summer. The days the patients did not answer the questionnaire were excluded from the analysis. We identified the most likely trigger for the patient’s asthma episodes. Once a reliable personalized model associating triggers with asthma episode is developed, it can be used to guide an action plan including preventive or remedial measures, as well as design targeted evaluations for better personalized care. IRB protocol defined 2 classes of users with access to patient data. The first class consisting of the physician and nurse who already had the patient’s clinical and personally identifiable information continued to have that access, in addition to the kHealth-collected data. The second class consisted of researchers who have access to the data collected by kHealth kit, but all privately identifiable data that clinician or nurse have access to was replaced by a serially chosen identifier. Specifically, the researchers also did not have access to the patient’s address or location. In fact, the gender, the weight, and the age information of the individual patient were intentionally deleted from the paper to remove personally identifiable data as stipulated in the approved IRB protocol.

### Winter to Spring

Patient A was diagnosed with severe asthma and monitored for 13 weeks, encompassing winter to spring 2018, and answered the questionnaire for 46 days, enough to identify the personalized triggers. The patient was prescribed albuterol and Atrovent (as rescue medication), as well as Dulera and Singulair (as controller medication). The patient took rescue medication for 24 days; experienced cough, wheeze, or chest tightness for 39 days; had limited activity for 26 days; showed abnormal PEF or FEV_1_ for 2 days; and was 15% adherent in taking controller medication. During the deployment period, PM2.5 was varying throughout, pollen was absent in the first half and present in the second half, and ozone was in a healthy range throughout. On the basis of the presence and absence of triggers in the patient’s environment, we calculated the probability of PM2.5 and pollen or PM2.5 alone being the trigger for the given symptom.

The patient experienced symptoms in the absence of pollen, indicating that PM2.5 is contributing to the patient’s asthma symptoms (Table 2). In the presence of pollen, the patient experienced higher number of severe symptoms such as chest tightness (chest tightness is more severe than wheeze [46]) and nighttime awakenings. The rescue medication intake was higher in the presence of pollen, which could have suppressed wheezing. For this patient, the presence of pollen or PM2.5 in the unhealthy range appears to be the primary contributing factor for asthma episodes. The combined presence of pollen and PM2.5 is associated with increased intensity of asthma episodes as evidenced by cumulative increase in number of symptoms. The pollen was in an unhealthy range for 20 days and PM2.5 for 41 days out of the 46 days the patient experienced asthma episodes. When validated with the clinician, the patient was identified to be allergic to pollen using the skin test. The ACT scores before and after the deployment confirmed that the patient’s asthma control was suboptimal.

Through continuous monitoring, we found that PM2.5 and pollen were the contributors to the patient’s asthma episodes, and the patient had poor adherence to controller medication. To improve asthma management, the intervention can be personalized by alerting the patient about high pollen and PM2.5 forecast. Furthermore, notification can be sent to improve the adherence to the controller medication. If asthma episodes recur even after being adherent toward controller medication, the clinician can intervene with a modified asthma action plan.

### Winter

Patient B was classified as having moderate asthma and monitored for 13 weeks in winter of 2017-2018. But data from only the first 9 weeks were available for analysis, as the patient did not answer for 4 weeks toward the end of the deployment. The patient was prescribed albuterol (as rescue medication) and Singulair (as controller medication) and answered the questionnaire for 50 days, of which the patient experienced asthma episodes for 45 days. Patient B experienced wheezing for 27 days, activity limitation for 15 days, nighttime awakenings on 1 day, took rescue medication for 24 days, had abnormal PEF or FEV_1_ values for 6 days, and was 50% adherent toward controller medication. Only PM2.5 was in an unhealthy range during the deployment period, whereas ozone was in a healthy range, and pollen was absent.

From Table 3, it can be observed that PM2.5 contributed to the patient’s wheeze and led to the intake of rescue medication. The patient did not experience symptoms on all the days the PM2.5 was in an unhealthy range because of patient’s adherence to controller medication, which was 50%. In consequence, the patient can be proactively notified when PM2.5 is forecast to be in the unhealthy range and may also be reminded to take prescribed medication. Of the 45 days the patient experienced asthma episodes, PM2.5 was in an unhealthy range for 40 days. To enhance asthma management, the patient can improve adherence toward controller medication and avoid exposure to PM2.5 when it is high. If this does not help, the patient should be reevaluated to adjust the asthma control plan. To identify the patient’s reaction to other triggers, the experiment needs to be repeated in other seasons.

### Fall

Patient C was diagnosed with moderate asthma and monitored for 5 weeks and 4 days in the fall of 2017. The patient answered the questionnaire 33 out of 36 days of deployment, of which the patient showed asthma episodes for 17 days. The patient was prescribed albuterol (as rescue medication), Symbicort and Singulair (as controller medications), and prednisone (oral steroid). The patient had cough and wheeze for 11 days, had activity limitation for 4 days, took rescue medication for 6 days, showed abnormal PEF or FEV_1_ for 9 days, and had an adherence of 63% toward controller medication. The outdoor environment remained uniform with respect to pollen, ozone, and PM2.5 throughout the deployment.

Higher intake of wheeze and rescue medication was observed in the initial stages of the deployment. Because pollen, PM2.5, and ozone were present throughout the deployment, all the 3 triggers appear to be contributing to the patient’s asthma symptoms (see Table 4, and we were not able to separate the triggers on the basis of the data we have). The patient also took oral steroids to control the asthma episodes, which reflected in the later part of the deployment by significant reduction in the number of asthma episodes. Of the 17 days the patient experienced asthma episodes, PM2.5 was in an unhealthy range for 13 days, pollen for 16 days, and ozone for 7 days. The kHealth framework has the potential to aid patient C in self-management of asthma by alerting the patient when pollen or PM2.5 can exacerbate asthma and reminding the patient to take medication to improve adherence. Furthermore, the clinician can also be notified when the patient takes the oral steroid, an indication of poor asthma control, to enable timely intervention. To exonerate some factors and identify triggers precisely, the experiment should be repeated in the season when pollen is absent.

### Summer

Patient D had mild asthma and was monitored for 4 weeks and 3 days in the summer of 2018. The patient was prescribed albuterol (as rescue medication) and Asmanex and Singulair (as controller medications). Patient D had answered the questionnaire for 29 out of 30 days and experienced asthma episodes for 6 days. The patient experienced cough, wheeze, chest tightness, or hard and fast breathing for 5 days; activity limitation and nighttime awakenings on 1 day; and the adherence toward the controller medication was 70%. The lung function measurements were normal throughout the study period. Out of the 6 days the patient suffered from asthma episodes, pollen was in an unhealthy range for 3 days, ozone for 4 days, and PM2.5 for 6 days. On the basis of observations, PM2.5 is suspected to be the major contributor, followed by ozone and pollen. However, this patient had sparse asthma episodes to identify the triggers precisely. In general, none of the patients deployed in summer had sufficient asthma episodes.

### Limitations

The objective of this study was to show the ability of kHealth kit to identify personalized triggers for each asthma patient and demonstrate the statistical significance of our findings for each patient across different seasons. However, to maximize participation in the study, all eligible pediatric patients who volunteered were enrolled on a first-come-first-serve basis to test the efficacy of continuous monitoring using low-cost sensors for asthma management. Therefore, the period of observation of patients did not necessarily coincide with season transitions. This turned out to be a limitation for contrasting the patient’s lung function and asthma episodes in allergy and nonallergy seasons. Furthermore, as the deployment was on a rolling basis, the patient deployment periods did not coincide. As such, we were unable to combine results from several patients for the same period and provide statistical significance. However, we are optimistic that we can repeat our experiments considering various allergy seasons and patients’ susceptibilities, especially given the 63% kit adherence that shows that this technology is acceptable to the patients and can monitor their asthma behavior in different seasons. Because of electrical interference, the data from the Foobot had to be excluded. Although the Fitbit data were reliable, the reason for reduced activity measured by Fitbit could be due to asthma or school routine or because Fitbit was not worn or powered. Hence, Fitbit did not provide originally envisioned insights in our analysis. Our observational study involved collection of the largest variety of PGHD (sensor data and mobile app answers) and environmental data, offering us the possibility of identifying likely, but not definitive, triggers for an individual patient. However, we are at best able to make inferences based on co-occurrence of symptoms and triggers and hesitate to make any claims about causality beyond saying these correlations provide a good basis for generating a hypothesis for a more extensive randomized controlled trial (RCT). Determining the true causes is beyond the scope of this paper.

### Conclusions

The infrequent clinical visits, as practiced by traditional health care protocol, are unable to provide timely feedback and enable intervention. Through continuous monitoring, kHealth kit can provide detailed insights to the clinician about the personalized triggers for asthma patients and their adherence toward the prescribed asthma control protocol. Specifically, for patients such as patient A whose deployment spanned 2 seasons, the kHealth kit suggested, with evidence, the relevant triggers along with the patient’s adherence to the prescribed asthma action plan. This can aid the clinician in tailoring the asthma control protocols for a patient, thereby leading to better asthma management. Furthermore, kHealth kit was able to capture triggers across different seasons, which was evident from the determination of the variety of personalized triggers for the patients chosen from different seasons.

### Future Work

We plan to repeat the observational trial for different seasons for each patient. Redeployment will be carried out for winter patients to discover potential triggers in other seasons and also for spring and fall patients to disambiguate among multiple triggers by exonerating some. In addition, 2 separate 1-month deployments in 2 different seasons (allergic and nonallergic) will be attempted again as the earlier experiments yielded sparse data, and 1-month deployments that straddle across 2 seasons will be avoided in future. Moreover, 25% (21/23) of the entire patient cohort did not experience any asthma episodes. Eventually, with the kHealth system, we expect to identify for each patient triggers across seasons that cause worsening of the patient’s asthma and thereby aid the clinician with insights about triggers and patient adherence for a personalized action plan.

This study has also helped us design a future study involving self-management and intervention. As Foobot was giving unreliable data, it will be discontinued in our future studies with a replacement that will provide a reliable measure of indoor air quality that is sensitive to secondhand smoke exposure. The Android questionnaire will be updated to disambiguate the reason for reduced activity as it could have been due to asthma or normal school routine without extracurriculars or because of the Fitbit not being worn or powered. The data from the Peak Flow meter proved to be reliable, and a clear distinction could be observed between asthmatic and nonasthmatic days. As the data sources for outdoor environmental data were also reliable, it will be continued. Android app questionnaire will be replaced with a chatbot to improve ease of use [47,48]. As to our future study, we are now working with the School of Medicine at the University of South Carolina that has access to a larger cohort of patients under the care of a larger number of clinicians and specialists. This study will provide a better foundation to formulate a hypothesis for an RCT, which is our next step.

## Appendix

Multimedia Appendix 1 Links to websites and demo videos.

Multimedia Appendix 2 The questionnaire used in the kHealth Asthma Android app.

Multimedia Appendix 3 Terms and definitions.

## Abbreviations

ACT: Asthma Control Test
DCH: Dayton Children’s Hospital
FEV1: forced expiratory volume in 1 second
IRB: institutional review board
NICHD: National Institute of Child Health and Human Development
NIH: National Institutes of Health
PEF: peak expiratory flow
PGHD: patient-generated health data
PM2.5: particulate matter with diameter less than 2.5 micrometers
RCT: randomized controlled trial

## References

[1] Centers for Disease Control and Prevention.

[2] Thomsen SF (2015). Genetics of asthma: an introduction for the clinician. Eur Clin Respir J.

[3] (2017). World Health Organization.

[4] Vernon MK, Wiklund I, Bell JA, Dale P, Chapman KR (2012). What do we know about asthma triggers? A review of the literature. J Asthma.

[5] Engelkes M, Janssens HM, de Jongste JC, Sturkenboom MC, Verhamme KM (2015). Medication adherence and the risk of severe asthma exacerbations: a systematic review. Eur Respir J.

[6] Morrison D, Mair FS, Yardley L, Kirby S, Thomas M (2017). Living with asthma and chronic obstructive airways disease: using technology to support self-management - An overview. Chron Respir Dis.

[7] Hui CY, Walton R, McKinstry B, Jackson T, Parker R, Pinnock H (2017). The use of mobile applications to support self-management for people with asthma: a systematic review of controlled studies to identify features associated with clinical effectiveness and adherence. J Am Med Inform Assoc.

[8] Tinschert P, Jakob R, Barata F, Kramer J, Kowatsch T (2017). The potential of mobile apps for improving asthma self-management: a review of publicly available and well-adopted asthma apps. JMIR Mhealth Uhealth.

[9] Barrett MA, Humblet O, Marcus JE, Henderson K, Smith T, Eid N, Sublett JW, Renda A, Nesbitt L, Van Sickle D, Stempel D, Sublett JL (2017). Effect of a mobile health, sensor-driven asthma management platform on asthma control. Ann Allergy Asthma Immunol.

[10] The Environmental Observation Web and its Service Applications within the Future Internet.

[11] Asthma Forecast.

[12] 12 CH, Huang C, Lian Z (2006). A ubiquitous warning system for asthma-inducement.

[13] Finkelstein J, Cabrera M, Hripcsak G (1998). Web-based monitoring of asthma severity: a new approach to ambulatory management.

[14] Yoon HJ, Ra HK, Salekin A, Kim J, Stone DJ, Kim S, Lee JM, Son SH, Stankovic JA (2016). AsthmaGuide: an asthma monitoring and advice ecosystem. IEEE Wireless Health.

[15] Anantharam P, Banerjee T, Sheth A (2015). Knowledge-Driven Personalized Contextual mHealth Service for Asthma Management in Children. 2015 IEEE International Conference on Mobile Services.

[16] Wiki Knoesis.

[17] Sheth A, Jaimini U, Thirunarayan K, Banerjee T (2017). Augmented personalized health: how smart data with IoTs and AI is about to change healthcare. RTSI.

[18] (2006). InformedHealth.org.

[19] (2019). Fitbit.

[20] (2019). Microlife.

[21] Pollen.com.

[22] AIRNow.

[23] Weather Underground.

[24] Schatz M, Sorkness CA, Li JT, Marcus P, Murray JJ, Nathan RA, Kosinski M, Pendergraft TB, Jhingran P (2006). Asthma Control Test: reliability, validity, and responsiveness in patients not previously followed by asthma specialists. J Allergy Clin Immunol.

[25] Truong M, Iniguez JL, Chouhou D, Dessange JF, Gendrel D, Chaussain M (1995). [Measurement of peak expiratory flow in young children: comparison of four portable equipments]. Arch Pediatr.

[26] Hernando Sastre V, García-Marcos L, Gómez García J, Faura Martínez U, Rubio Pérez J, Navarro Ortiz MD, Hernández Dólera JA (2000). [Peak expiratory flow rate in 4- to 15-year-old children. Comparison of 3 measuring models]. An Esp Pediatr.

[27] Trasande L, Thurston GD (2005). The role of air pollution in asthma and other pediatric morbidities. J Allergy Clin Immunol.

[28] Murat C (2017). South Dakota State University.

[29] Tulure F (2017). Asthma trends in Mississippi coastal region with air pollutants and meteorological factors. Austin J Allergy.

[30] de Zambotti M, Goldstone A, Claudatos S, Colrain IM, Baker FC (2018). A validation study of Fitbit Charge 2™ compared with polysomnography in adults. Chronobiol Int.

[31] Paul SS, Tiedemann A, Hassett LM, Ramsay E, Kirkham C, Chagpar S, Sherrington C (2015). Validity of the Fitbit activity tracker for measuring steps in community-dwelling older adults. BMJ Open Sport Exerc Med.

[32] Jaimini U, Banerjee T, Romine W, Thirunarayan K, Sheth A, Kalra M (2017). Investigation of an Indoor Air Quality Sensor for Asthma Management in Children. IEEE Sens. Lett.

[33] Google.

[34] Sridharan V (2018). Sensor Data Streams Correlation Platform for Asthma Management.

[35] McQuaid EL, Kopel SJ, Klein RB, Fritz GK (2003). Medication adherence in pediatric asthma: reasoning, responsibility, and behavior. J Pediatr Psychol.

[36] Seth A, Anantharam P, Thirunarayan K (2014). kHealth: Proactive Personalized Actionable Information for Better Healthcare.

[37] Merchant R, Inamdar R, Henderson K, Barrett M, Su JG, Riley J, Van Sickle D, Stempel D (2018). Digital health intervention for asthma: patient-reported value and usability. JMIR Mhealth Uhealth.

[38] Bender B, Wamboldt FS, O'Connor SL, Rand C, Szefler S, Milgrom H, Wamboldt MZ (2000). Measurement of children's asthma medication adherence by self report, mother report, canister weight, and Doser CT. Ann Allergy Asthma Immunol.

[39] Frey U, Brodbeck T, Majumdar A, Taylor DR, Town GI, Silverman M, Suki B (2005). Risk of severe asthma episodes predicted from fluctuation analysis of airway function. Nature.

[40] National Asthma Education and Prevention Program.

[41] Harkins MS, Fiato K, Iwamoto GK (2004). Exhaled nitric oxide predicts asthma exacerbation. J Asthma.

[42] Pollen.com.

[43] EPA AIRNow.

[44] Calhoun WJ, Dick EC, Schwartz LB, Busse WW (1994). A common cold virus, rhinovirus 16, potentiates airway inflammation after segmental antigen bronchoprovocation in allergic subjects. J Clin Invest.

[45] Jaimini U, Thirunarayan K, Kalra M, Venkataraman R, Kadariya D, Sheth A (2018). "How Is My Child's Asthma?" Digital phenotype and actionable insights for pediatric asthma. JMIR Pediatr Parent.

[46] Walker HK, Hall WD, Hurst JW (1990). WheezingAsthma. Clinical Methods: The History, Physical, and Laboratory Examinations. 3rd edition.

[47] Kadariya D, Venkataramanan R, Yung YH, Kalra M, Thirunarayanan K, Sheth A (2019). kBot: Knowledge-enabled Personalized Chatbot for Asthma Self-Management.

[48] Sheth A, Yip HY, Iyengar A, Tepper P (2019). Cognitive services and intelligent chatbots: current perspectives and special issue introduction. IEEE Internet Comput.

