# Supplementary material for: Determination of Personalized Asthma Triggers From Multimodal Sensing and a Mobile App: Observational Study
Source: JMIR Pediatr Parent. 2019 Jun 27;2(1):e14300. doi: 10.2196/14300 (PMC6716491; doi:10.2196/14300)
Supplement: Multimedia Appendix 1 [file pediatrics_v2i1e14300_app1.docx]

**Appendix 1: Links to websites and demo videos.**

<http://wiki.knoesis.org/index.php/KHealth:_Semantic_Multisensory_Mobile_Approach_to_Personalized_Asthma_Care#Publications>

kHealthDash Demo video- <https://www.youtube.com/watch?v=yUgXCPwc55M>
