# Supplementary material for: Determination of Personalized Asthma Triggers From Multimodal Sensing and a Mobile App: Observational Study
Source: JMIR Pediatr Parent. 2019 Jun 27;2(1):e14300. doi: 10.2196/14300 (PMC6716491; doi:10.2196/14300)
Supplement: Multimedia Appendix 2 [file pediatrics_v2i1e14300_app2.docx]

**Appendix 2: The questionnaire from kHealth Asthma Android application**

| **Question** | **Answer type** |
| --- | --- |
| Did you experience any of the asthma symptoms? | Cough, Wheeze, Chest tightness, Hard and Fast breathing, Can’t talk in full sentences, Nose opens wide |
| How many times did you take *[name of the rescue inhaler prescribed]* inhaler today due to asthma symptoms? | 1,2,3,4,5,6+ |
| Did you take *[name of the controller medication prescribed]* today? | Yes, No |
| How much did asthma symptoms limit your activity? | None, A little, Most of the day, At least half of the day |
| Did you wake up last night due to asthma symptoms? | Yes/No |
