# Supplementary material for: Determination of Personalized Asthma Triggers From Multimodal Sensing and a Mobile App: Observational Study
Source: JMIR Pediatr Parent. 2019 Jun 27;2(1):e14300. doi: 10.2196/14300 (PMC6716491; doi:10.2196/14300)
Supplement: Multimedia Appendix 3 [file pediatrics_v2i1e14300_app3.docx]

**Appendix 3: Terms and definitions**

1. *PM2.5 occurrence:* PM2.5(Particulate Matter 2.5) value greater than 50, threshold provided by EPA. This data source and threshold has already been used in Trasande, Leonardo, and George D. Thurston. "The role of air pollution in asthma and other pediatric morbidities." Journal of allergy and clinical immunology 115, no. 4 (2005): 689-699.
2. *Ozone occurrence:* Ozone value greater than 50, threshold provided by EPA. This data source and threshold has already been used in Trasande, Leonardo, and George D. Thurston. "The role of air pollution in asthma and other pediatric morbidities." Journal of allergy and clinical immunology 115, no. 4 (2005): 689-699.
3. *Pollen occurrence:* Pollen value greater than 2.4, threshold provided by pollen.com. This data source and threshold has already been used in Kececi, Murat Cagatay. "Monitoring Pollen Counts and Pollen Allergy Index Using Satellite Observations in East Coast of the United States." (2017).
4. *Symptom occurrence:* Days the patient experienced any asthma symptom
5. *Night-time awakenings:* Days the patient woke up at night due to asthma symptom. There is no specific threshold as it a boolean value.
6. *Asthma episodes*: Refers to occurences of some or all of the following:
   1. all the 6 symptoms collected from the Android app questionnaire: cough, wheeze, chest tightness, hard and fast breathing, can’t talk in full sentences, nose opens wide
   2. Activity limitation (collected from Android app questionnaire)
   3. Night-time awakenings (collected from Android app questionnaire)
   4. Rescue medication intake (collected from Android app questionnaire)
   5. Abnormal PEF/FEV1 values (from Peak Flow meter)
7. *Active sensing:* the part of data collection, which requires the patient to be actively involved such as responding to Android app questionnaire and taking Peak Flow meter readings.
8. *Passive sensing:* The data collection from Fitbit, Foobot and outdoor web services, which do not require active patient involvement.
